# Antibiotic Resistance and Virulence Gene Patterns Associated with Avian Pathogenic *Escherichia coli* (APEC) from Broiler Chickens in Qatar

**DOI:** 10.3390/antibiotics10050564

**Published:** 2021-05-11

**Authors:** Alreem Johar, Najlaa Al-Thani, Sara H. Al-Hadidi, Elyes Dlissi, Mahmoud H. Mahmoud, Nahla O. Eltai

**Affiliations:** 1 Research and Development Department, Barzan Holdings, Doha 7178, Qatar; ajohar@barzanholdings.com (A.J.); nalthani@barzanholdings.com (N.A.-T.); 2 Biomedical Research Center, Microbiology Department, Qatar University, Doha 2713, Qatar; s.alhadidi@qu.edu.qa; 3 Al-Asayl Veterinary Laboratory, Microbiology Unit, Doha 2713, Qatar; dlissi@yahoo.com; 4 Ministry of Municipality and Environment, Doha 22332, Qatar; mhmahmoud@mme.gov.qa

**Keywords:** Avian pathogenic *E. coli*, APEC, virulence genes, antibiotics, resistance, AMR

## Abstract

Avian Pathogenic *Escherichia coli* (APEC) is the contributing agent behind the avian infectious disease colibacillosis, which causes substantial fatalities in poultry industries that has a significant impact on the economy and food safety. Several virulence genes have been shown to be concomitant with the extraintestinal survival of APEC. This study investigates the antibiotic resistance patterns and APEC-associated virulence genes content in *Escherichia coli* isolated from non-healthy and healthy broiler chickens from a commercial poultry farm in Qatar. A total of 158 *E. coli* strains were isolated from 47 chickens from five different organs (air sac, cloacal, kidney, liver, and trachea). Based on genetic criteria, 65% were APEC strains containing five or more virulence genes, and 34% were non-pathogenic *E. coli* (NPEC) strains. The genes *ompT*, *hlyF*, *iroN*, *tsh*, *vat*, *iss*, *cvi*/*cva*, and *iucD* were significantly prevalent in all APEC strains. *E. coli* isolates showed 96% resistance to at least one of the 18 antibiotics, with high resistance to ampicillin, cephalothin, ciprofloxacin, tetracycline, and fosfomycin. Our findings indicate high antibiotic resistance prevalence in non-healthy and healthy chicken carcasses. Such resistant *E. coli* can spread to humans. Hence, special programs are required to monitor the use of antibiotics in chicken production in Qatar.

## 1. Introduction

The poultry sector in Qatar remains invested in promoting food sustainability in the country. The nation has been striving to increase the production of products that farmers can produce locally, such as meat, fresh vegetables, fish, poultry, and dairy products [1]. Unfortunately, different challenges, including diseases, have resulted in considerable losses in this segment. *Escherichia coli* (*E. coli*) are harmless, normal inhabitants of the gastrointestinal tract of animals and human beings [2]. Despite this, researchers have found that *E. coli* can cause various illnesses that might affect individuals of all ages [3]. Specific strains of *E. coli* can invade multiple birds’ organs, causing peritonitis, perihepatitis, air sacculitis, pericarditis, and other extraintestinal infections [4]. Collectively, experts have termed extraintestinal conditions as colibacillosis. Avian pathogenic *E. coli* (APEC) is a causative agent for colibacillosis.

The pathogenic capability of *E. coli* is mainly due to multiple virulence factors. The presence of at least five or more of eight virulence-associated genes determines the presence of APEC [5]. These genes include iron acquisition genes (*iucD*, *iroN*), adhesion genes (*tsh*), toxin genes (*vat*, *hlyF*), serum resistance genes (*iss*), housekeeping proteases (*ompT*), and ColV Operon genes (*cvi*/*cva*). Some avian and human extraintestinal pathogenic *E. coli* (ExPEC) tend to have comparable phylogenic backgrounds and share similar virulence genes [6]. Moreover, similarities between the APEC strain O1:K1: H7, neonatal meningitis *E. coli* (NMEC), and human uropathogenic *E. coli* (UPEC) were discovered [6]; hence, there is a high probability of horizontal gene transfer between APEC and phylogenetically similar human ExPEC strains. APEC continues to cause havoc in the poultry segment by being a leading cause of colibacillosis with undesirable health outcomes. Antibiotics are an essential combative technique that farmers use against APEC and other pathogens [7].

For decades, farmers in the poultry business have been using antibiotics for multiple purposes as a form of therapy or growth promoters [8,9]. One of the repercussions of using antibiotics in poultry farming has been a surge in antibiotic-resistant bacteria that ultimately affect humans [10].

Fundamentally, APEC is potentially transmissible to humans and potentially causes various health hazards. Despite scientists not categorizing *E. coli* as pathogens, these bacteria have been identified as opportunistic pathogen [11], and humans acquire these pathogens mainly through the fecal–oral route. *E. coli*, in general, can cause gastrointestinal diseases in both food-production animals and, consequently, humans [12]. A high incidence of antibiotic resistance was reported among *E. coli* isolated from poultry farms in Qatar [13]. Antibiotic resistance in APEC strains escorted by compatibility of horizontal gene transfer between APEC strains and human ExPEC strains is hugely worrying as cases of resistant APEC infection in humans may be related to the gene transfers.

Poultry farming is a crucial segment in the Qatari economy, especially during Qatar’s blockade and the COVID-19 pandemic period. Regrettably, the outbreak of antibiotic-resistant diseases could have a catastrophic impact on this industry [14]. This research is important in understanding the prevalence of antibiotic resistance among APEC strains, as well as the relationship between antibiotic use and the spread of antibiotic resistance. However, minimal publications have been published in Qatar, mainly by our group, to evaluate the level of antibiotic resistance in the veterinary sector. This deficiency has been cited by the World Health Organization “Joint External Evaluation of Qatar: Mission Report” [15], and recommendations were made to enhance and support an active antimicrobial resistance program in animals. Moreover, previous studies of APEC have focused on various organs [5], namely, air sacs, cloaca, kidney, liver, and trachea, which have been chosen to study in parallel to provide a clearer image of the manifestation of APEC within the avian system. Hence, this study aims to identify APEC isolated from different organs, namely air sacs, cloacal, kidney, liver, and trachea samples of non-healthy and healthy broiler chickens, profiling their antibiotic resistance phenotypically and genotypically, and identifying virulence genes associated with them.

## 2. Results

### 2.1. Sample Isolation

In total, 158 *Escherichia coli* (*E. coli*) were isolated. Of these, 135 (85%) were from non-healthy chickens: from air sac samples (21), cloacal samples (27), kidney samples (29), liver samples (28), and trachea samples (30). The remaining 23 (15%) were from healthy chickens: from air sac samples (3), cloacal samples (11), kidney samples (2), liver samples (2), and trachea samples (5).

### 2.2. Detection of APEC Associated Virulence Genes in Non-Healthy and Healthy Chicken Samples

**Figure 1:**
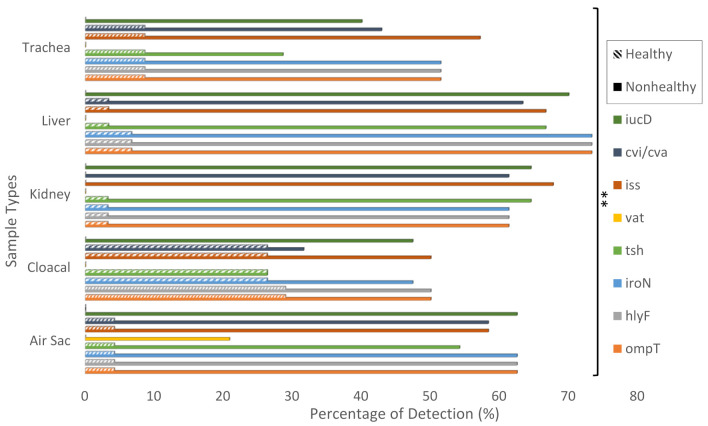
Percentage of APEC associated virulence genes detected in isolated air sac, cloacal, kidney, liver, and trachea samples of non-healthy and healthy chickens. Multiplex PCR was performed from non-healthy and healthy chicken samples of different sample types (air sac, cloacal, kidney, liver, and trachea). The PCR reactions’ amplification products were run on a 1% agarose gel at 150 V for 80 min. The gels were then scored for the presence or absence of the APEC virulence genes. ** indicates significance (*p*-value < 0.01).

To better understand the circulating virulent strains’ diversity, we further evaluated the APEC isolated from both non-healthy and healthy chickens. Based on the genetic criteria for the pathogenicity, isolates containing at least five virulence genes were considered APEC strains, and isolates containing less than five virulence genes were considered non-pathogenic *E. coli* (NPEC) strains (Figure S1). Out of the 158 *E. coli* isolates, 103 (65%) were classified as Avian Pathogenic *Escherichia coli* (APEC) strains; 86% from non-healthy chickens and 14% from healthy chickens and 55 (35%) were classified as non-pathogenic *E. coli* (NPEC) strains; 85% from non-healthy chickens and 15% from healthy chickens. For APEC samples, all 16 air sac samples were obtained from non-healthy chickens, 29 from cloacal samples (19 from non-healthy chickens and 10 from healthy chickens), 18 from kidney samples (all from non-healthy chickens), 22 from liver samples (21 from non-healthy chickens and 1 from a healthy chicken), and 18 from trachea samples (15 from non-healthy chickens and 3 from healthy chickens). Of the 103 APEC strains, 5 contained all 8 virulence genes, 56 contained 7 virulence genes, 30 contained 6 virulence genes, and 11 contained only 5 virulence genes. The virulence genes were detected at the following percentages in APEC and NPEC, respectively: *ompT* (99%, 16%), *hlyF* (99%, 16%), *iroN* (97%, 16%), *tsh* (81%, 11%), *vat* (5%, 0%), *iss* (97%, 16%), *cvi*/*cva* (89%, 4%), and iucD (81%, 1%). The detection of virulence genes in APEC strains was significantly more than in NPEC strains (*p*-value < 0.01). Alternatively, the virulence genes were detected at the following percentages in non-healthy and healthy chickens, respectively: *ompT* (69%, 78%), *hlyF* (69%, 78%), *iroN* (68%, 74%), *tsh* (54%, 70%), *vat* (4%, 0%), *iss* (70%, 65%), *cvi*/*cva* (59%, 65%), and iucD (65%, 0%). The detection of virulence genes in non-healthy chicken samples was found to be significantly more than in healthy chicken samples of all types: air sac, cloacal, kidney, liver, and trachea (*p*-value < 0.01) (Figure 1).

### 2.3. Sequence Analysis of Virulence Genes Detected in Isolated APEC Strains

Sequence analysis of amplified *ompT*, *hlyF*, *iroN*, *iss*, and iucD PCR products aligned to the sequences with respective genes found in a known APEC strain at 99% similarity, “*E. coli* O1:H7 strain CH138 plasmid pECCH138-1, the complete sequence “ [16] (accession number: DQ381420.1).

### 2.4. Clustering Analysis Based on Genetic Profile of E. coli Isolated from Non-Healthy and Healthy Chickens

**Figure 2:**
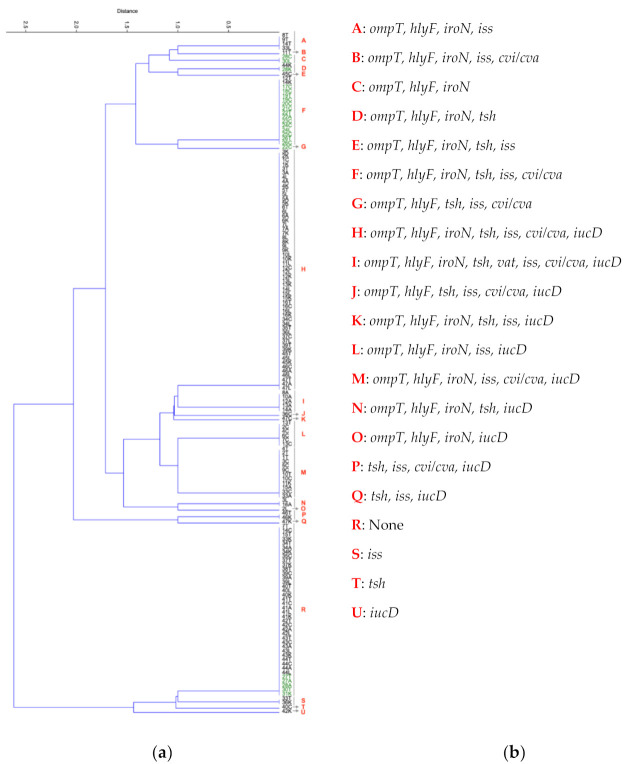
Agglomerative hierarchical algorithm illustrating the similarity amongst *E. coli* isolates from air sac, cloacal, kidney, liver, and trachea samples isolated from non-healthy and healthy chicken samples based on the presence and absence of APEC associated virulence genes. (**a**) The distance (*y*-axis) represents how closely related the clustered samples are relative to each other. Samples in black are from non-healthy chickens and samples in green are from healthy chickens. Numbers indicate chicken sample ID. Sample types are labeled as Air sac (A), cloacal (C), Kidney (K), Liver (L), and Trachea (T). The samples are clustered as follows. In cluster A (8T, 9T, 14T, 33L). In cluster B (11T), In cluster C, (28C, 30L). In cluster D, (44K, 28K). In cluster E, (45C). In cluster F, (12T, 14K, 17C, 18C, 19T, 19C, 20C, 21C, 22T, 22A, 23C, 24C, 24L, 25C, 26T, 26C). In cluster G, (22C). In cluster H, (3K, 2K, 1C, 1K, 3T, 3A, 4L, 4A, 4K, 5T, 5L, 5A, 5K, 6T, 6L, 6A, 6K, 7L, 7A, 7K, 8L, 8K, 9L, 9K, 10L, 10K, 11L, 12C, 12L, 12K, 13L, 13K, 14L, 15L, 15K, 16T, 16C, 16L, 16K, 34C, 34L, 35T, 36L, 37C, 37L, 39T, 39K, 45T, 45L, 45K, 46C, 46A, 46L, 47T, 47A, 47L). In cluster I, (8A, 10A, 12A, 13A, 14A). In cluster J, (36C). In cluster K, (47C). In cluster L, (13T, 2C, 4C, 6C, 7C, 13C). In cluster M, (4T, 2T, 1T, 3C, 5C, 9C, 10T, 10C, 11K, 15A, 33C, 33A). In cluster N, (3L, 16A). In cluster O, (2L). In cluster P, (46T, 46K). In cluster Q, (47K). In cluster R, (7T, 14C, 15T, 33K, 34T, 34A, 34K, 35C, 37T, 37K, 38T, 39C, 39A, 39L, 40T, 40L, 40K, 41T, 41C, 41A, 41L, 41K, 42T, 42C, 42A, 42L, 43T, 43C, 43A, 43L, 43K, 44T, 44C, 44A, 44L, 27T, 27A, 28A, 30T, 31K). In cluster S, (33T, 36K). In cluster T, (40C). In cluster U, (42K). (**b**) The APEC associated virulence genes linked to each cluster.

Based on clustering analysis (Figure 2), cluster H was found to be the largest cluster with all 56 samples containing the following genetic profiles: *ompT*, *hlyF*, *iroN*, *tsh*, *iss*, *cvi*/*cva*, and iucD, indicating that all non-healthy chicken samples in cluster H are classified as APEC strains. In addition, it is essential to note that virulence genes are typically detected in groups of three or more as clusters A-Q which all contain genetic profiles of three or more genes, account for 72% of the samples and cluster R which contains none of the virulence genes accounts for 25% of the samples with clusters S, T, and U accounting for only four samples with only one virulence gene. This tells us that the detection of one gene means it is very likely to detect other genes. A closer look at clustering patterns of healthy chicken samples shows their occurrence in cluster R with no virulence genes, cluster C (three virulence genes) and cluster D (four virulence genes), all indicating healthy NPEC chicken samples. APEC strains from healthy chicken samples were all found in cluster F (six virulence genes) and cluster G (five virulence genes) and were either cloacal or trachea samples except for two samples in cluster F from the air sac and liver.

### 2.5. Restriction Fragment Length Polymorphism (RLFP) Analysis

RLFP analysis of clusters (Figure S2) revealed three uniform fragment patterns for all samples with HaeIII except for non-healthy chicken liver sample 2L (Lane 4) and non-healthy chicken trachea sample 8T (Lane 11), which showed only two fragments. Sample 2L is part of cluster O and is the only sample in the cluster with the genetic profile *ompT*, *hlyF*, *iroN*, and *iucD*, and sample 8T is part of cluster A with genetic profile *ompT*, *hlyF*, *iroN*, and *iss* (Figure S2). Both samples contain only four virulence genes which could contribute to their shared fragmentation patterns.

### 2.6. Phenotypic Resistance Profile of E. coli Isolated from Non-Healthy and Healthy Chickens

**Figure 3:**
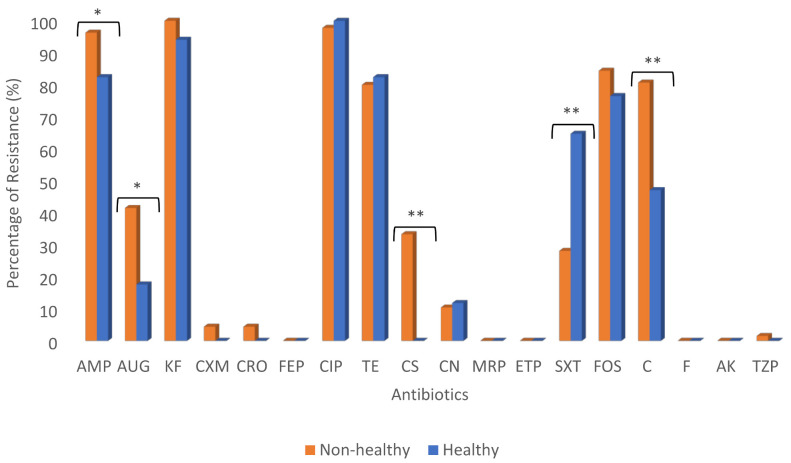
Antimicrobial resistance profile of *E. coli* isolates from non-healthy chicken (*n* = 135) and healthy chicken (*n* = 17). Isolates were tested against 18 clinically relevant antibiotics (Liofilchem^®^, Roseto degli Abruzzi, Italy) with exception of colistin, where Etest was used. Phenotypically resistance *E. coli* isolates were then analyzed using Microsoft Office Excel 2010. AMP, Ampicillin; AUG, Amoxicillin/Clavulanic acid, KF, Caphalothin; CXM, Cefuroxime; CRO, Ceftriaxone; FEP, Cefepime; CIP, Ciprofloxacin; T.E., Tetracycline; C.S., Colistin; C.N., Gentamicin; MRP, Meropenem; ETP, Ertapenem; SXT, Trimethoprim-sulphamethoxazole; FOS, Fosfomycin; C, Chloramphenicol; F, Nitrofurantoin; A.K., Amikacin; TZP, Piperacillin/Tazobactam. * indicates significance (*p*-value < 0.05) and ** indicates significance (*p*-value < 0.01).

The percentage of antimicrobial resistance profile of *E. coli* isolates obtained from non-healthy (*n* = 135) and healthy (*n* = 17) chickens are summarized in Figure 3 and Table 1. Generally, *E. coli* isolates of both non-healthy and healthy chickens showed relatively high resistance to ampicillin (100%, 82.4%), cephalothin (100%, 94.1%94), ciprofloxacin (97.8%, 100%), tetracycline (80.3%, 82.4%), and fosfomycin (84.4%, 76.5%), respectively. Moderate resistance was reported against amoxicillin/clavulanic acid (41.5%, 17.6%) in *E. coli* isolates of non-healthy and healthy chickens, respectively. On the other hand, lower resistance was recorded against gentamicin (10.4%, 11.8%) in non-healthy and healthy chickens, respectively. Furthermore, lower resistance was recognized against cefuroxime (4.4%), ceftriaxone (4.4%), and piperacillin/tazobactam (1.5%) among *E. coli* isolates of non-healthy chickens. In comparison, these antibiotics were utterly susceptible in isolates of healthy chickens (Figure 3). Percentage of resistance to trimethoprim-sulphamethoxazole and nitrofurantoin were significantly different between *E. coli* isolates from non-healthy and healthy chickens. Furthermore, a relatively high colistin resistance percentage (33.3%) showed only among *E. coli* isolated from non-healthy chickens. Nearly 1.5% of *E. coli* from non-healthy chicken (*n* = 2) were resistant to at least three antibiotics, whereas 2.2%, 10.4%, 34.1%, 31.1%, 14.1%, 3.0%, and 3.7% showed resistance to four, five, six, seven, eight, nine, and ten antibiotics, respectively. In contrast, 4.3% of the *E. coli* isolated from healthy chickens (*n* = 1) were resistant to at least two antibiotics, while 8.7%, 4.3%%, 30.4%, 21.7%, and 4.3% of isolates were resistant to three, five, six, seven, and eight antibiotics, respectively. The resistance of *E. coli* isolates of both non-healthy and healthy chickens was recorded to be highest at six antibiotics (34.1%, 30.4%), respectively. Six *E. coli* isolates (3.8%) were classified as Extended-Spectrum Beta-Lactamase (ESBL) producers, and all six isolates were obtained from non-healthy chickens. Furthermore, 99.3% were multidrug-resistant (MDR) (Table 1). Multidrug resistance is defined as an isolate showing resistance to at least one agent in three or more antimicrobial categories [17]. As demonstrated in Figure 3, antimicrobial drug resistance of ampicillin, amoxicillin/clavulanic acid, colistin, trimethoprim-sulphamethoxazole, and chloramphenicol showed a statically significant difference between *E. coli* isolates of non-healthy and healthy chickens.

### 2.7. Genotypic Resistance Profile of E. coli Isolated from Non-Healthy and Healthy Chickens

**Figure 4:**
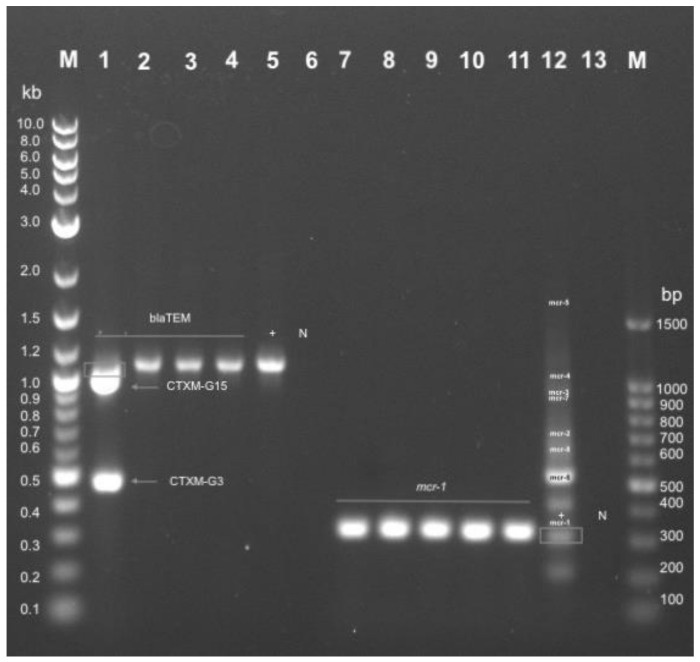
Detection of antimicrobial resistance (AMR) genes in phenotypically resistant *E. coli* from air sac, cloacal, kidney, liver, and trachea samples of non-healthy chickens. Representative samples are shown for Multiplex PCR performed to detect ESBL related AMR Genes (bla_(TEM, CTXM-G15, CTXM-G3)_) and colistin resistance-related AMR genes (*mcr*-1, *mcr*-2, *mcr*-3, *mcr*-4, *mcr*-5). The amplification products of the ESBL gene PCR reactions and Colistin resistance genes PCR reactions were loaded respectively for one sample and were loaded in one lane and were run on a 1.2% agarose gel at 180 V for 50 min. In Lane 1, non-healthy chicken trachea sample (2T). In Lane 2, non-healthy chicken trachea sample (5T). In Lane 3, non-healthy chicken trachea sample (15T). In Lane 4, non-healthy chicken cloacal sample (13C). In Lane 5, bla_TEM_ positive PCR control. In Lane 6, bla_TEM_ negative PCR control. In Lane 7, non-healthy chicken liver sample (5L). In Lane 8, non-healthy chicken trachea sample (5T). In Lane 9, non-healthy chicken kidney sample (6K). In Lane 10, non-healthy chicken air sac sample (6A). In Lane 11, non-healthy chicken liver sample (6L). In Lane 12, *mcr*-(1-7) positive PCR control. In Lane 13, *mcr*-(1-7) negative PCR control. M; molecular size (weight) marker. Bp; base pair.

The detection of antimicrobial resistance genes of *E. coli* isolates obtained from non-healthy chickens are presented in Figure 4 (representative samples are shown). Samples showing phenotypic ESBL characteristics (*n* = 6) confirmed corresponding genotypic ESBL profiles by detecting the bla_TEM_ gene in all samples (Lane 1-4; Figure 4). Additionally, one of the ESBL samples contained both the bla_CTXM-G15_ and bla_CTXM-G3_ genes (Lane 1; Figure 4). Five (83%) of the confirmed ESBL samples were classified as APEC. Samples showing phenotypic colistin resistance (*n* = 44) confirmed corresponding genotypic colistin resistance profiles by detecting the *mcr-*1 gene in all samples (Lane 7-11; Figure 4). Thirty-two (73%) of the confirmed colistin-resistant samples were classified as APEC.

## 3. Discussion

A recent study in Qatar has shown the abuse of antibiotics in the poultry industry, which has resulted in the detection of antibiotic resistance genes in *E. coli* isolated from broiler chickens [18]. The rise of antibiotic-resistant strains and mainly multidrug and colistin resistance strains in Qatar’s poultry farms can only result in unfavorable outcomes for poultry handlers and consumers. Detection of a high number of resistant APEC strains in commercial poultry farms is especially alarming. APEC is now known to belong to ExPEC groups, which could facilitate its infection to humans. There is currently no data on phenotypic antibiotic resistance profiles of APEC strains or molecular-based studies for detection of APEC-associated virulence genes in avian colibacillosis diseased chickens in Qatar. Therefore, this study has aimed to identify APEC isolated from different organs of non-healthy and healthy broiler chickens with genotypic and phenotypic profiling of antibiotics and the genetically identifying virulence genes associated with APEC.

In this study, the frequency of eight virulence genes and their disease pathology roles was evaluated amongst non-healthy and healthy chickens. Non-healthy chickens were selected due to suspicion of infection by APEC based on physical morbidities and symptoms. Criteria for identifying APEC strains were set at detecting at least five or more virulence genes, enhancing APEC pathogenicity [5]. In this study, we found that APEC-virulence genes *ompT*, *hlyF*, *iroN*, *tsh*, *iss*, and *cvi*/*cva* were significantly detected in APEC and NPEC among cloacal samples from healthy chickens and with less frequency in trachea samples (Figure 1). This finding correlates to previous studies on APEC-virulence genes reporting these genes’ presence to be endemic in the avian microbiome, classifying them as Avian Fecal *E. coli* (AFEC) [19], explaining their high detection rate in cloacal samples of healthy chickens. However, the danger lies in their presence in other avian organs. Significant detection of APEC-virulence genes in APEC and NPEC in trachea samples from healthy chickens relates to APEC transmission via fecal–oral route. Nevertheless, due to their low levels of detection in other organs (liver, kidney, and air sac), it can be concluded that APEC did not spread in these healthy chickens. Furthermore, APEC virulence-associated genes *ompT*, *hlyF*, *iroN*, *tsh*, *iss*, and *cvi*/*cva* were found more frequently in APEC from non-healthy chicken samples with higher frequency in liver and kidney samples followed by cloacal then trachea samples and lastly air sac samples (Figures S1 and S2). The high detection rate of these genes in liver and kidney samples indicates the widespread of APEC in these chickens as they could reach internal organs, namely, the liver and kidney. Regarding the other APEC-associated virulence genes, *vat* was only significantly found in APEC from air sac samples of non-healthy chickens with no detection in other APEC or NPEC samples from non-healthy or healthy chickens. Alternatively, *iucD* was only significantly detected in non-healthy chicken samples (96% APEC; 4% NPEC) with no detection in any healthy chicken samples (Figure 1). The high frequency of *ompT* (99%), *hlyF* (99%), *iroN* (97.1%), *tsh* (80.6%), *iss* (97.1%), *cvi*/*cva* (89.3%) and *iucD* (80.6%) among APEC strains compares to previous studies which have also found these set of genes to be present in APEC cases. Subedi et al. (2018) [4] found similar percentages of APEC-associated virulence genes in broiler chickens in Nepal. The detection rates of virulence genes in APEC strains in this study were: *ompT* (100%), *hlyF* (100%), *iroN* (100%), *tsh* (62.2%), *iss* (100%), *cvi*/*cva* (57.8%), and *iucD* (97.8%). In another study, Kwon et al., (2008) [20] found similar percentages of virulence genes among 18 APEC strains which were: *iss* (100%), *tsh* (94%), *iucD* (83%), and *cvi*/*cva* (16%).

Clustering analysis reveals the presence of outbreak APEC strain indicated by Cluster H (Figure 2). This is supported through sequence analysis and previous publications corresponding to similar APEC outbreak stain O1:H7 [16]. Cluster F and G containing APEC strains from healthy chickens, the natural condition of the avian microbiome, and APEC transmission via the fecal–oral route can account for the cloacal and trachea samples. Nonetheless, detecting APEC strain in healthy air sac and liver samples can indicate APEC presence and early onset of avian colibacilloses in chickens considered physically healthy.

This study reported a high percentage of resistance, 96%, to at least one antibiotic among *E. coli* isolated from both non-healthy and healthy chicken samples. Additionally, high resistance of 96.3% and 82.4% to ampicillin, 100% and 94.1% to cephalothin, 97.8% and 100% to ciprofloxacin, 80% and 82.4% to tetracycline, 84.4% and 76.5% to fosfomycin was reported among non-healthy and healthy chickens, respectively. In addition, 99.3% of the isolates were identified as MDR that renders treatment options with antibiotics. A similar study conducted in Bangladesh [21] reported 100% resistance to ampicillin and tetracycline among MDR *E. coli* isolated from broiler chickens. This is in agreement with our findings. In another study from northern Egypt, a similar result was detected by Moawad et al. [22], who observed high resistant *E. coli* isolates from raw chicken meat with different antibiotics and a lower percentage of MDR. In their study, the phenotypic antibiotic resistance profile of *E. coli* isolates to ampicillin, tetracycline, streptomycin, trimethoprim/sulphamethoxazole, and cefotaxime were 71.4%, 80.9%, 61.9%, 61.9%, and 33.3%, respectively. However, none of the *E. coli* isolates were resistant to colistin, which differs from our findings. This study’s most worrying finding is the high percentage of *E. coli* isolates from non-healthy chickens (33.3%) resistant to the last-resort antibiotic colistin. This type of resistance was detected among *E. coli* isolates from both imported and locally produced chickens and broiler chickens from Qatar [18] and might be related to colistin use on chicken farms. The *mcr-*1 gene was found in all isolates that exhibited colistin resistance, demonstrating its role in colistin resistance. Several international studies have investigated the phenotypic resistance profile of *E. coli* isolates from chicken, such as those conducted in Saudi Arabia [23], Algeria [24], China [25], Canada [26], and the Netherlands [27]. Our results showed a high resistance profile to the first-line therapeutic antibiotics that are commonly used in poultry disease treatment like ampicillin and tetracycline [28]. Personal communication with the poultry source farm owner revealed the use of multiple antibiotics, including colistin, amoxicillin, enrofloxacin, and fosfomycin. These antibiotics revealed significant resistance among *E. coli* isolates in our study as we reported 28.5%, 37.3% [29], 94.3%, and 80.4%, respectively.

This study reported a significantly high percentage of MDR *E. coli* (99.3%) from chicken samples. In alignment, we have previously detected high MDR resistant *E. coli* prevalence in other food chain studies; 33% in broiler chickens [18] and 27% in healthy food handlers [17]. In contrast, the prevalence of ESBL-producing *E. coli* was 3.8% in this study, which is relatively low compared to other resistant percentages. However, a similar result was observed by a previous study among retail chicken, in which 4.2% of the isolates were ESBL-producing *E. coli* [13]. Most third-generation cephalosporins are not used in the poultry industry; they are used only intravenously at healthcare facilities to manage human patients with serious infections. Proper control and monitoring are required to decrease the high percentage of antibiotic resistance in Qatar’s poultry farms.

## 4. Materials and Methods

### 4.1. Sample Collection

Research approval to process samples was obtained from Qatar University’s Institutional Biohazard Committee under approval number QU (QU-IBC-2018/034). Forty-seven chickens were obtained from one of Qatar’s big commercial farms for four months between September and December 2020, including 32 non-healthy chickens and 15 healthy chickens. Clinical assessment was conducted initially by a veterinary doctor to differentiate between non-healthy and healthy chickens suspected of having APEC. Houses registering an increase in the mortality rate, with birds having low body weight, presenting depression, loss of appetite, no water drinking, and disinclination to move were considered non-healthy and suspected cases of APEC infection. Field autopsy on some dead birds mainly showed fibrinopurulent aerosaculitis, perihepatitis, and pericarditis (peritonitis were rare), further emphasizing the suspicion of APEC infection. Chickens that did not display these conditions were considered healthy. The non-healthy and healthy chickens were then euthanized. Samples were collected from 5 different organs, namely, air sacs, cloacal, kidney, liver, and trachea of each of the 47 chickens. Of these in total we manage to isolate 247 *E. coli*. A maximum aseptic technique was observed during the collection of these samples that were saved in sterile tubes. The collected samples were appropriately labeled, then transported in cooled boxes (4–8 °C) to Qatar University Biomedical Research Center laboratories (Doha, Qatar). Upon arrival, samples were stored at 24 °C and processed within 24 h. Tracheal and air sac samples were transferred within transport media, whereas cloacal, kidney, and liver samples were collected and stored in airtight containers.

### 4.2. Bacterial Isolation

A small portion of the collected air sacs (200 µL), trachea (200 µL), liver (300 mg), kidneys (300 mg), and feces (10 mg) were suspended in 3 mL tryptic soy broth (TSB). The samples were then incubated overnight at 37 °C. After incubation, 10 µL of culture were streaked onto a selective medium CHROMagar *E. coli* plate (Himedia, India) and incubated at 37 °C for 24 h. Single typical *E. coli* colonies (green color with a smooth surface) were randomly selected and subsequently streaked onto MacConkey agar plates (Liofilchem^®^, Roseto degli Abruzzi, Italy), and incubated at 37 °C for 24 h, followed by streaking on nutrient agar (Remel, ThermoFisher Scientific, Lenexa, KS, USA). Then, Indole positive isolates for lactose fermentation were presumptively identified as *E. coli* and confirmed biochemically with a Biomic V3 system (Giles Scientific, Santa Barbara, CA, USA) with the Crystal Enteric/Nonfermenter ID kit (BBL, B.D., Sparks, MD, USA) and preserved at −80 °C until further analysis.

### 4.3. Identification of Avian Pathogenic E. coli (APEC) Strains

PCR was used to differentiate non-pathogenic and avian pathogenic *E. coli* based on APEC-associated virulence genes. First, DNA was extracted from pure cultures of *E. coli* using QIAamp^®^ UCP pathogen miniKit (QIAGEN, Hilden, Germany) following the manufacturer’s instructions and then used to run a multiplex PCR assays targeting 8 virulence genes (namely, *ompT*, *hlyF*, *iroN*, *tsh*, *vat*, *iss*, *cvi*/*cva*, and *iucD* associated with APEC using previously published primers [30]. A detailed description of the PCR condition is summarized in supplementary Tables S1 and S2. Amplified products were subjected to electrophoresis in 1.2% agarose (Agarose-LE, Ambion^®^, Pittsburgh, PA, USA), stained with ethidium bromide (Promega, Madison, WI, USA), and visualized using iBright CL 1000 system (Invitrogen, Waltham, MA, USA).

### 4.4. Clustering of Virulence Genes

An agglomerative hierarchical algorithm was used to derive a cluster analysis dendrogram from establishing the relationship between the *E. coli* isolates of non-healthy and healthy chicken samples and their corresponding sample types (air sac, cloacal, kidney, liver, and trachea) based on the presence and absence of the 8 APEC associated virulence genes (*ompT*, *hlyF*, *iroN*, *tsh*, *vat*, *iss*, *cvi*/*cva*, and *iucD*). The scores ’1’ and ‘0’ were given for the presence and absence of bands, respectively. The data obtained by scoring the different sets of virulence genes’ genetic profiles were subjected to cluster analysis. A hierarchical cluster dendrogram was created using Past software version 4.03. Similarity matrix values were used for cluster analysis [31].

### 4.5. Sequencing and Sequence Analysis

Amplicon products that were obtained from PCR reactions targeting APEC associated virulence genes were purified by ExoSAP-IT (G.E. Healthcare life science, Chicago, IL, USA) according to the manufacturer instructions and then subjected to sequencing reactions using specific forward and reverse primers for each gene with Big Dye Terminator Reaction Mix (Applied Biosystems, Waltham, MA, USA). The reaction products were purified using Big Dye XTerminator purification Kit (Applied Biosystems) per manufacturer instructions and run on ABI 3500 XL sequencer (Fisher scientific, Pittsburgh, PA, USA). The sequencing of the virulence gene amplicons was confirmed using the online NCBI blast tool.

### 4.6. Phenotypic Antibiotic Susceptibility Testing

*E. coli* isolates were screened for resistance to 18 antibiotics. Antibiotic susceptibility testing was performed using the standard Kirby-Bauer (K.B.) disk except for colistin, where the E-test method was used, according to the Clinical and Laboratory Standards Institute (CLSI) [32] recommendations and guidelines. Standard strains, *E. coli* ATCC^®^ 25,922 was used as control organisms for antimicrobial drug susceptibility testing. The 18 clinically relevant antibiotics used to screen the antibiotic susceptibility of *E. coli* were chosen because they are widely used by the panel at Hamad Medical Corporation in Qatar, as they are commonly relevant to Gram-negative bacteria.

### 4.7. Extended Spectrum Beta Lactomase (ESBL) Phenotype Confirmation

ESBL phenotypic resistance was confirmed using the double-disc synergy test (DDST) as described previously by CLSI [33]. Briefly, 20/10 μg discs of amoxicillin-clavulanate and 30 μg discs of ceftazidime and ceftriaxone (B.D.-Sensi Disc™), placed onto Mueller–Hinton agar (Oxoid Ltd., Basingstoke, Hampshire, UK) inoculated with a microbial suspension of 0.5 McFarland turbidity at a distance of 15 mm apart from the edge of the amoxicillin-clavulanate disc. The cefoxitin (30 μg, B.D.-Sensi Disc™) disc was placed in any available space remaining on the plate. Extension of the edge of the exhibition zone by greater than 5 mm towards the amoxicillin-clavulanate disc, together with cefoxitin’s susceptibility, was interpreted as positive for ESBL production.

Briefly, synergy was determined between a 20/10 μg disc of amoxicillin-clavulanate (B.D.-Sensi Disc™) and 30 μg disc of ceftazidime and ceftriaxone (B.D.-Sensi Disc™).

### 4.8. Genotypic Characterization of Colistin and ESBL Resistant Isolates

Total DNA was extracted from colistin-resistant isolates as described above. The multiplex PCR was performed as described elsewhere using previously published primers to screen for the most common genes, namely, *mcr-1*, *mcr-2*, *mcr-3*, *mcr-4*, and *mcr-5*, which are responsible for horizontal transfer of colistin resistance among *E. coli* [34]. The PCR mixture contained 0.5 μM concentrations of each pair of primers, 30 ng of DNA, 12.5 μL of master mix (Hot star Taq Plus), 1× of Corolload load concentrate (CoralLoad, QIAGEN, Hilden, Germany), and DEPC-treated water up to a volume of 25 μL. The reactions were amplified in Thermal Cycler as follows: denaturation at 95 °C for 15 min; 25 cycles of 95 °C for 30 min, 58 °C for 1.5 min, and 72 °C for 1 min; and a final extension at 72 °C for 10 min.

Total DNA extracted from *E. coli* isolates that were tested positive for ESBL by DDST were investigated for *bla*_TEM_, *bla*_SHV,_ and *bla*_CTX-M-G (1,2,8,9,25,3,14,15)_ genes via PCR using previously published primers [35,36]. For more details on the PCR condition, see Tables S3 and S4. Amplified products were subjected to electrophoresis in 1.2% agarose (Agarose-LE, Ambion^®^, Pittsburgh, PA, USA), stained with ethidium bromide (Promega, Madison, WI, USA), and visualized using Bio-Rad gel doc system (Bio-rad, Gel Doc tm X.R. System 170–8170, Montréal, QC, Canada).

### 4.9. Restriction Fragment Length Polymorphism (RFLP) Analysis

Based on the clustering patterns, RFLP was performed using one sample from each cluster. Before RLFP, the 16S rRNA region was amplified using the universal bacterial primers (27F: 5′-AGAGTTTGATYMTGGCTCAG-3′ and 1492R: 5′-GGTTACCTTGTTACGACTT-3′, both to a final concentration of 0.5 mM [37], 25 µL of Hot Star Taq master mix (QIAGEN, Hilden, Germany), ≤20 ng of DNA and RNase free water up to 50 µL. The reaction was amplified in Biometra TAdvanced Thermal Cycler (Analytik, Jena, Germany) as previously described [38]. Restriction enzymes used were *ECOR*I, *BG*II, *BG*III, *Hinf*I, *Alu*I, *Hae*III, *Hinc*II, *Hind*III, *Sac*I, *Sma*I (Takara, Japan) according to instructions of the manufacturer. The RLFP reactions were carried out in 10 µL volume containing 0.5 µL enzyme, 1 µL buffer, 2.5 µL, an additional 1 µL Bovine Serum Albumin (BSA) for *Sma*I and nuclease-free water up to 10 µL. RLFP samples were then incubated at 37 °C except for *Sma*I samples, which were incubated at 30 °C. After incubation, 1 µL of stop solution was added to the samples, which were then subjected to electrophoresis in 1.2% agarose (Agarose-LE, Ambion^®^, Pittsburgh, PA, USA), stained with ethidium bromide (Promega, Madison, WI, USA), and visualized using Bio-Rad gel doc system (Bio-rad, Gel Doc tm X. R. System 170–8170, Montréal, QC, Canada).

### 4.10. Data Analysis

Data entry, initial analysis, and figure design were done using Microsoft Office Excel 2010 (Microsoft Corporation, New York, NY, USA) to generate figures and run initial analysis. Further statistical analysis was performed using Graph Pad version 8 (San Diego, CA, USA), and PAST software version 4.03 (Oslo, Norway) was used for clustering analysis. The chi-square test was calculated using Pearson probability value (*p*-value) to compare the antibiotic resistance and virulence gene patterns between non-healthy and healthy chicken samples. A *p*-value less than 0.05 was considered statistically significant.

## 5. Conclusions

This is the first study in Qatar to genetically identify Avian Pathogenic *Escherichia coli* (APEC) and its resistance to relevant antibiotics among broiler chickens. It is also one of the few studies that have set out to simultaneously detect APEC strains in several avian organs. Our findings confirm the presence of APEC strains among broiler chickens within poultry farms as well as high antibiotic resistance prevalence in non-healthy and healthy chickens. Such resistant *Escherichia coli* (*E. coli*) could easily spread to humans through chicken meat consumption and non-compliance with hygiene practices among farmworkers. Such studies are very important, considering the rapid growth of poultry farms in Qatar, to overcome the blockade imposed since June 2017 and the need to reach self-sustenance. This should also be paralleled with studying resistance patterns along the food chain, including farmworkers and food handlers. Thus, special programs are required to regulate the use of antibiotics in Qatar’s poultry sector and monitor the spread and transmission of APEC within the poultry industry.

## Figures and Tables

**Table 1:** Antimicrobial resistance profile of *E. coli* isolates from non-healthy (*n* = 135) and healthy (*n* = 23) chickens.

Resistance Phenotype ^1^	Multi-Drug Resistance (MDR) ^2^	Frequency		Percentage (%)	
		*E. coli* (NH) ^3^	*E. coli* (H) ^4^	*E. coli* (NH) ^3^	*E. coli* (H) ^4^
AMP, K.F., CIP, T.E., FOS, C	+	20.0	1.0	14.8	5.9
AMP, AUG, K.F., CIP, T.E., FOS, C	+	17.0	1.0	12.6	5.9
AMP, K.F., CIP, TE., C.S., FOS, C	+	9.0	0.0	6.7	0.0
AMP, AUG, K.F., CIP, T.E., C.S., FOS, C	+	8.0	0.0	5.9	0.0
AMP, AUG, K.F., CIP, T.E., SXT, FOS, C	+	6.0	0.0	4.4	0.0
AMP, K.F., CIP, FOS, C	+	5.0	0.0	3.7	0.0
AMP, K.F., CIP, T.E., SXT, FOS, C	+	4.0	2.0	3.0	11.8
AMP, K.F., CIP, T.E., C.S., C	+	4.0	0.0	3.0	0.0
AMP, K.F., CIP, T.E., C.S., FOS	+	4.0	0.0	3.0	0.0
AMP, K.F., CIP, T.E., SXT, FOS	+	3.0	4.0	2.2	23.5
AMP, AUG, K.F., CIP, FOS, C	+	3.0	0.0	2.2	0.0
AMP, K.F., CIP, T.E., SXT, C	+	2.0	2.0	1.5	11.8
AMP, AUG, K.F., CIP, T.E., CSC.S., SXT, FOS	+	2.0	0.0	1.5	0.0
AMP, AUG, K.F., CIP, T.E., FOS	+	2.0	0.0	1.5	0.0
AMP, AUG, K.F., CIP, T.E., SXT, C	+	2.0	0.0	1.5	0.0
AMP, AUG, K.F., T.E., C.S., FOS, C	+	2.0	0.0	1.5	0.0
AMP, K.F., CIP, C.S., FOS, C	+	2.0	0.0	1.5	0.0
AMP, K.F., CIP, C.S., SXT, FOS, C	+	2.0	0.0	1.5	0.0
AMP, K.F., CIP, SXT, FOS, C	+	2.0	0.0	1.5	0.0
AMP, K.F., CIP, T.E., FOS	+	2.0	0.0	1.5	0.0
MP, K.F., CXM, CRO, CIP, T.E., C.N., SXT, FOS, C	+	2.0	0.0	1.5	0.0
AMP, AUG, KFKF, CIP, T.E., C.N., FOS	+	1.0	1.0	0.7	5.9
AMP, KF, CIP, TE, CN, SXT, FOS, C	+	1.0	1.0	0.7	5.9
AMP, K.F., CIP, T.E., SXT	+	1.0	1.0	0.7	5.9
AMP, AUG, K.F., CIP, SXT, FOS, C, TZP	+	1.0	0.0	0.7	0.0
AMP, AUG, K.F., CIP, C	+	1.0	0.0	0.7	0.0
AMP, AUG, K.F., CIP, FOS, C	+	1.0	0.0	0.7	0.0
AMP, AUG, K.F., CIP, SXT, C	+	1.0	0.0	0.7	0.0
AMP, AUG, K.F., CIP, SXT, FOS	+	1.0	0.0	0.7	0.0
AMP, AUG, K.F., CIP, T.E., C	+	1.0	0.0	0.7	0.0
AMP, AUG, K.F., CIP, T.E., CSC.S., C.N., FOS, C	+	1.0	0.0	0.7	0.0
AMP, AUG, K.F., CIP, T.E., CSC.S., C.N., FOS, C, TZP	+	1.0	0.0	0.7	0.0
AMP, AUG, K.F., CIP, T.E., C.N., SXT, C	+	1.0	0.0	0.7	0.0
AMP, AUG, K.F., CIP, T.E., FOS	+	1.0	0.0	0.7	0.0
^5^ AMP, AUG, K.F., CXM, CRO, CIP, T.E., C.N., SXT, C	+	1.0	0.0	0.7	0.0
^5^ AMP, AUG, K.F., CXM, CRO, CIP, T.E., C.S., FOS, C	+	1.0	0.0	0.7	0.0
AMP, K.F., CIP	+	1.0	0.0	0.7	0.0
AMP, K.F., CIP, C.S., C	+	1.0	0.0	0.7	0.0
AMP, K.F., CIP, FOS	+	1.0	0.0	0.7	0.0
AMP, K.F., CIP, SXT	+	1.0	0.0	0.7	0.0
AMP, K.F., CIP, SXT, FOS	+	1.0	0.0	0.7	0.0
AMP, K.F., CIP, T.E., C	+	1.0	0.0	0.7	0.0
AMP, KF, CIP, TE, CN, SXT, FOS	+	1.0	0.0	0.7	0.0
AMP, K.F., CIP, T.E., C.S., C.N., C	+	1.0	0.0	0.7	0.0
AMP, K.F., CIP, T.E., C.S., C.N., SXT, FOS, C	+	1.0	0.0	0.7	0.0
AMP, K.F., CIP, T.E., C.S., SXT, FOS, C	+	1.0	0.0	0.7	0.0
^5^ AMP, K.F., CXM, CRO, CIP, T.E.	+	1.0	0.0	0.7	0.0
^5^ AMP, K.F., CXM, CRO, CIP, T.E., C.S., FOS, C	+	1.0	0.0	0.7	0.0
AMP, K.F., T.E., SXT, FOS	+	1.0	0.0	0.7	0.0
AUG, K.F., CIP, T.E., C.S., FOS, C	+	1.0	0.0	0.7	0.0
K.F., CIP, C.N.	+	1.0	0.0	0.7	0.0
K.F., CIP, C.N., FOS	+	1.0	0.0	0.7	0.0
K.F., CIP, C.N., FOS, C	+	1.0	0.0	0.7	0.0
K.F., CIP, FOS	+	0.0	2.0	0.0	11.8
AMP, AUG, K.F., CIP, SXT, FOS, C	+	0.0	1.0	0.0	5.9
^6^ CIP, T.E.	-	0.0	1.0	0.0	5.9

^1^ AMP, Ampicillin; AUG, Amoxicillin/Clavulanic acid, K.F., Cephalothin; CXM, Cefuroxime; CRO, Ceftriaxone; FEP, Cefepime; CIP, Ciprofloxacin; TE., Tetracycline; C.S., Colistin; C.N., Gentamicin; MRP, Meropenem; ETP, Ertapenem; SXT, Trimethoprim-sulphamethoxazole; FOS, Fosfomycin; C, Chloramphenicol; F, Nitrofurantoin; A.K., Amikacin; TZP, Piperacillin/Tazobactam. ^2^ All intermediates were considered as susceptible; 99.3% of resistant isolates found to be MDR. ^3^ Isolated from non-healthy chickens. ^4^ Isolated from healthy chickens. ^5^ ESBL: extended spectrum beta lactamase producer; ESBL isolates (*n* = 6). ^6^ Non-MDR profile.

## Data Availability

The data presented in this study are available on request from the corresponding author.

## Supplementary Materials

The following are available online at https://www.mdpi.com/article/10.3390/antibiotics10050564/s1, Figure S1. Detection of virulence genes among APEC isolated from non-healthy and healthy chickens; Figure S2: Restriction Fragment Length Polymorphism (RLFP) using HaeIII restriction enzyme on select *E. coli* isolates from non-healthy and healthy chicken samples (*n* = 21) amongst sample types; Table S1: PCR component used for the identification of Avian Pathogenic *E. coli* (APEC) strains.; Table S2. PCR conditions conducted in Biometra TAdvanced Thermal Cycler; Table S3. PCR component used for the genotypic characterization of colistin and ESBL resistant isolates; Table S4. PCR conditions conducted in Biometra TAdvanced Thermal Cycler (Analytik, Jena, Germany) to test for positive ESBL producing E. coli strains.
