# Supplementary material for: Antibiotic Resistance and Virulence Gene Patterns Associated with Avian Pathogenic Escherichia coli (APEC) from Broiler Chickens in Qatar
Source: Antibiotics (Basel). 2021 May 11;10(5):564. doi: 10.3390/antibiotics10050564 (PMC8151107; doi:10.3390/antibiotics10050564)
Supplement: Supplementary file 1 [file antibiotics-10-00564-s001.zip › antibiotics-1194274-supplementary.pdf]

Supplementary Materials: The following are available online at www.mdpi.com/2079-6382/10/5/564/s1, Figure S1: Figure S1. Detection of virulence genes among APEC isolated from non-healthy and healthy chickens; Figure S2: Restriction Fragment Length Polymorphism (RLFP) using HaeIII restriction enzyme on select *E. coli* isolates from non-healthy and healthy chicken samples (*n* = 21) amongst sample types; Table S1: PCR component used for the identification of Avian Pathogenic *E. coli* (APEC) strains.; Table S2. PCR conditions conducted in Biometra TAdvanced Thermal Cycler; Table S3. PCR component used for the genotypic characterization of colistin and ESBL resistant isolates; Table S4. PCR conditions conducted in Biometra TAdvanced Thermal Cycler (Analytik, Jena, Germany) to test for positive ESBL producing E. coli

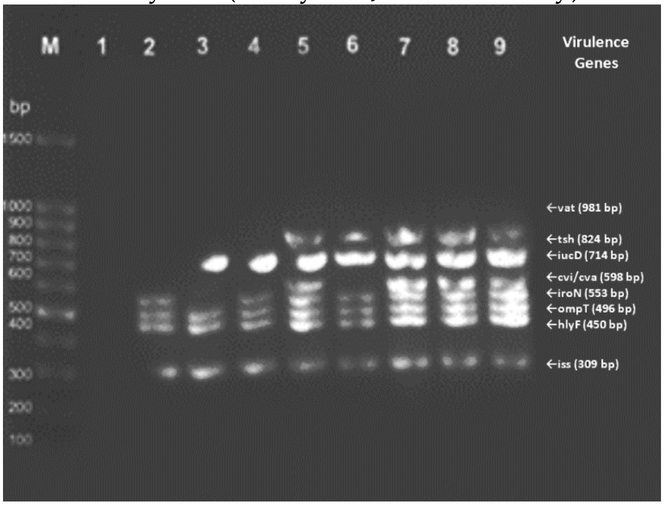

Figure S1. Detection of virulence genes among APEC isolated from non-healthy and healthy chickens. Representative samples are shown for Multiplex PCR performed to detect APEC associated virulence genes (*ompT*, *hlyF*, *iroN*, *tsh*, *vat*, *cvi*/*cva* and *iss*) and Uniplex PCR performed to detect APEC associated virulence gene (*iucD*). The amplification products of all PCR reactions for one sample were loaded in one lane and were run on a 1% agarose gel at 150 V for 80 min. In Lane 1, healthy chicken trachea sample (30T). In Lane 2, non-healthy chicken trachea sample (16T). In Lane 3, non-healthy chicken cloacal sample (6C). In Lane 4, non-healthy chicken cloacal sample (16C). In Lane 5, non-healthy chicken liver sample (8L). In Lane 6, non-healthy chicken cloacal sample (17C). In Lane 7, non-healthy chicken liver sample (13L). In Lane 8, non-healthy chicken liver sample (15L). In Lane 9, non-healthy chicken air sac sample (12A). M; molecular size (weight) marker. Bp; base pair.

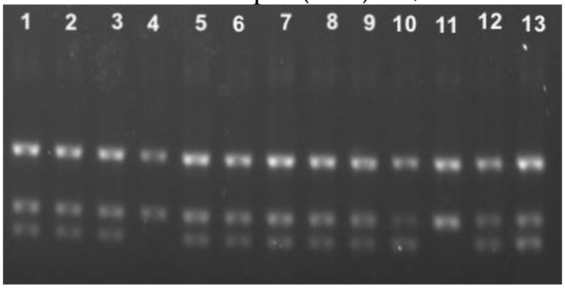

Figure S2. Restriction Fragment Length Polymorphism (RLFP) using HaeIII restriction enzyme on select *E. coli* isolates from non-healthy and healthy chicken samples (*n* = 21) amongst sample types (air sac, cloacal, kidney, liver, and trachea). Representative samples are shown for the RLFP reaction using the HaeIII restriction enzyme. Samples for RLFP were selected based on clustering patterns following statistical analysis. One sample from each cluster was selected (*n* = 21). Following the 16S PCR reaction, sample amplicons were incubated for 1 h at 37 °C after the addition of HaeIII restrictions enzyme and reaction buffer. After the incubation period, 1 μL stopping dye was added, and samples were loaded on

1.2% agarose gel and run for 50 min at 180 V. In Lane 1, healthy chicken kidney sample (28K). In Lane 2, healthy chicken cloacal sample (28C). In Lane 3, non-healthy liver sample (3L). In Lane 4, non-healthy chicken liver sample (2L). In Lane 5, healthy chicken cloacal sample (22C). In Lane 6, non-healthy chicken kidney sample (14K). In Lane 7, healthy chicken cloacal sample (26C). In Lane 8, non-healthy chicken air sac sample (6A). In Lane 9, non-healthy chicken air sac sample (12A). In Lane 10, non-healthy chicken kidney sample (11K). In Lane 11, non-healthy chicken trachea sample (8T). In Lane 12, non-healthy chicken cloacal sample (2C). In Lane 13, non-healthy chicken trachea sample (11T).

Table S1. PCR component used for the identification of Avian Pathogenic *E. coli* (APEC) strains.

PCR Component	Volume (μL)
HotStarTaq® Plus master mix (QIAGEN, Germany)	10
0.5 mM forward primer	1.0
0.5 mM reverse primer	1.0
1× of Corolload load concentrate (CoralLoad, QIAGEN)	2.0
DNA template	1.5
Nuclease free water (QIAGEN, Germany)	Up to 20
Total	20

Table S2. PCR conditions conducted in Biometra TAdvanced Thermal Cycler (Analytik, Jena, Germany) to differentiate between non-pathogenic and avian pathogenic *E. coli* based on APEC-associated virulence genes.

Primer	PCR Cycling Condition
ompT	94 °C for 5 min; 30 cycles of 30 s at 94 °C, 1 min at 60 °C, and 2 min at 68 °C; and 72 °C for 7 min.
hlyF	94 °C for 5 min; 30 cycles of 30 s at 94 °C, 1 min at 60 °C, and 2 min at 68 °C; and 72 °C for 7 min.
iroN	94 °C for 5 min; 30 cycles of 30 s at 94 °C, 1 min at 60 °C, and 2 min at 68 °C; and 72 °C for 7 min.
tsh	94 °C for 5 min; 30 cycles of 30 s at 94 °C, 1 min at 54 °C, and 1 min at 72 °C; and 72 °C for 7 min.
vat	94 °C for 5 min; 30 cycles of 30 s at 94 °C, 1 min at 54 °C, and 1 min at 72 °C; and 72 °C for 7 min.
iss	94 °C for 5 min; 30 cycles of 30 s at 94°C, 30 s at 58 °C, and 3 min at 68 °C; and 72 °C for 7 min.
cvi/cva	94 °C for 5 min; 30 cycles of 30 s at 94°C, 30 s at 58 °C, and 3 min at 68 °C; and 72 °C for 7 min.
iucD	94 °C for 5 min; 30 cycles of 1 min at 94 °C, 1 min at 55 °C, and 1 min at 72 °C; and 72 °C for 7 min.

Table S3. PCR component used for the genotypic characterization of colistin and ESBL resistant isolates.

PCR Component	Volume (μL)
HotStarTaq® Plus master mix (QIAGEN, Germany)	10
0.5 mM forward primer	1.0
0.5 mM reverse primer	1.0

1× of Corolload load concentrate (CoralLoad, QIAGEN)	2.0
50 ng DNA template	1.5
Nuclease free water (QIAGEN, Germany)	Up to 20
Total	20

Table S4. PCR conditions conducted in Biometra TAdvanced Thermal Cycler (Analytik, Jena, Germany) to test for positive ESBL producing *E. coli* strains.

Primer	PCR Cycling Condition
[bla_{TEM} and bla_{SHV}]	96 °C for 5 min.; 32 cycles of 30 s at 96 °C, annealing at 44 °C (bla_{TEM}) and 58 °C (bla_{SHV}) for 45 s, following 60 s at 72 °C and then at 72 °C for 10 min.
[$bla_{CTX-M-G\ (1,2,8,9,25)}$]	95 °C for 2 min.; 30 cycles of 25 s at 95 °C, 40 s at 52 °C, following 50 s at 72 °C and then 6 min at 72 °C.
[$bla_{CTX-M-G\ (3,\ 14)}$]	95 °C for 5 min; 30 cycles of 25 s at 95 °C, 40 s at 66 °C, following 50 s at 72 °C and then 6 min at 72 °C.
[$bla_{CTX-M-G15}$]	95 °C for 5 min; 30 cycles of 30 s at 95 °C, 30 s at 55 °C, following 30 s at 72 °C and then 5 min at 72 °C.
